# Unexpected finding of T-cell lymphoma in a previously healthy 16-year-old patient after a thorax trauma: a case report

**DOI:** 10.1186/1752-1947-8-371

**Published:** 2014-11-18

**Authors:** Anna Bach Okholm-Hansen, Stig Brorson

**Affiliations:** 1 University of Copenhagen, Nørregade 10, 1165 Copenhagen, Denmark; 2 Department of Orthopedic Surgery, Herlev University Hospital, Herlev Ringvej 75, 2730 Herlev, Denmark

## Abstract

**Introduction:**

We describe the clinical course and emphasize the difficulties in diagnosing T-cell lymphoblastic lymphoma. The differential diagnostic difficulties have previously been described in regard to pneumonia, but to the best of the authors’ knowledge this is the first case report to describe lymphoma in relation to trauma.

**Case presentation:**

A previously healthy 16-year-old Danish boy presented to our hospital with chest pain and accentuated protruding thoracic veins. Ten days prior to hospitalization he had suffered a blunt thoracic trauma while playing soccer. After drainage of an excessive amount of pleural fluid, he developed severe respiratory distress. A chest tube was inserted and he was transferred to a level 1 trauma centre. Here, a computed tomography scan unexpectedly revealed significantly swollen mediastinal and retroperitoneal lymph nodes, and he was later diagnosed with T-cell lymphoblastic lymphoma.

**Conclusions:**

This case emphasizes the importance of reacting to an unexplained large amount of pleural fluid after a patient suffers thoracic trauma and to consider possible underlying causes. This report is mainly addressed to emergency personnel, but it is also relevant to pediatricians, surgeons, anesthesiologists, and general practitioners.

## Introduction

T-cell lymphoblastic lymphoma is a rare type of aggressive non-Hodgkin lymphoma, with approximately 15 cases reported per year in Denmark. This condition usually affects male adolescents, who often present with pleural effusion and varying degrees of B-symptoms and respiratory symptoms. In very rare cases superior vena cava syndrome and drumstick fingers can be seen. The etiology is associated with exposure to radiation, pesticides, or congenital or acquired immunosuppression. It arises from immature T-cells, which infiltrate nodal structures or extra nodal structures such as the bone marrow, spleen, or central nervous system. One-third of the tumors have translocations involving the alpha and delta T-receptor loci, resulting in T-cell receptor promoter and enhancer elements, and various transcription factors and high expression of these in precursor thymocytes. T-cell lymphoblastic lymphoma is treated with chemotherapy regimens such as CHOP (cyclophosphamide, hydroxydaunorubicin, oncovin and prednisolone), similar to those used for acute lymphoblastic leukimia. With current treatment, the five-year survival rate is between 80 and 90% in children and between 45 and 55% in adults. Early diagnosis and treatment of both the respiratory symptoms and the underlying disease is crucial in order to improve the prognosis of this potentially life-threatening condition [1,2].

## Case presentation

**Figure 1:**
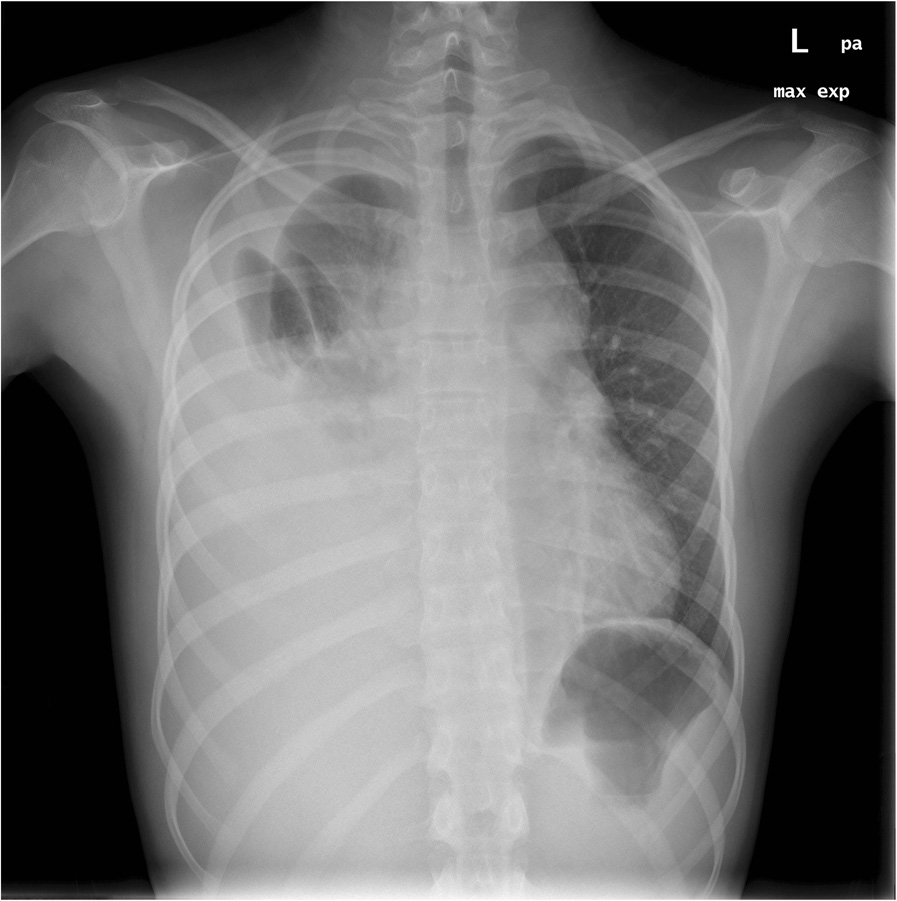
**X-ray of thorax.** Right side atelectasis, pleural effusion, and broadened mediastinum.

A previously healthy 16-year-old Danish boy presented to our emergency department with diffuse chest pain. Ten days prior to hospitalization he had suffered a blunt trauma to the chest while playing soccer. Closer inspection revealed accentuated vein drawing on the front of the chest and slight direct and indirect tenderness of the thorax. During lung auscultation, ceased respiration sounds on the right side and normal vesicular respiration on the left side were found. A chest X-ray revealed a collapsed right lung and pleural effusion (Figure 1). He was clinically unaffected and the vital parameters were normal.

A pleural drain was established and 2.5L of clear yellow fluid was drained. During the following few hours he went into respiratory distress (saturation 85% on 15L of oxygen), became hypotensive (blood pressure 64mmHg over 40mmHg) and tachypneic (respiratory frequency 24 breaths/minute). We administered 2L of saline solution and continuous positive airway pressure, which briefly stabilized the patient. On suspicion of lung embolism, an acute echocardiography was performed. This revealed pericardial effusion and dilatation of the right atrium. His respiratory and circulatory function became increasingly unstable. He was intubated and transferred to a level 1 trauma centre.

**Figure 2:**
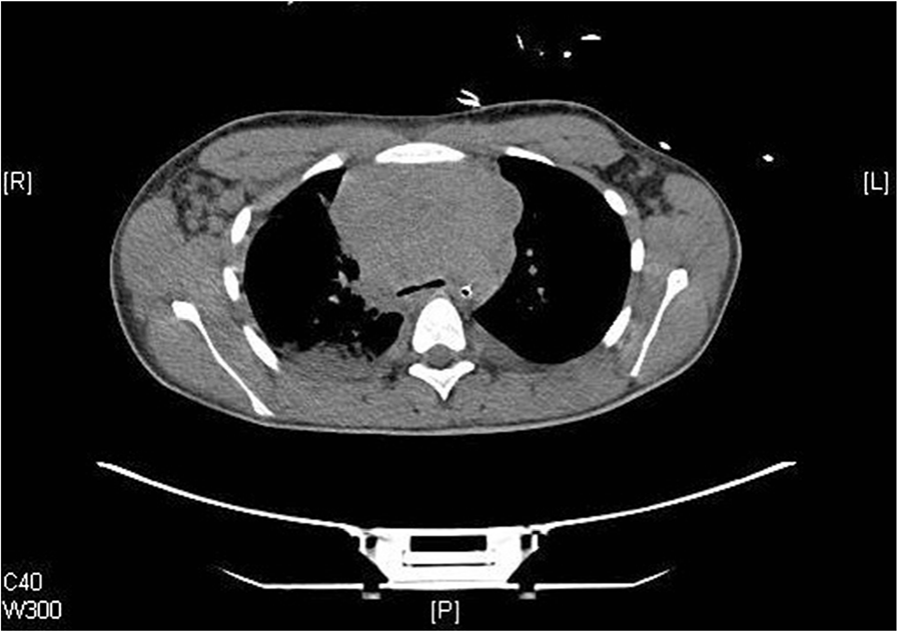
**Computed tomography scan of mediastinum.** Massive mediastinal tumor mass.

**Figure 3:**
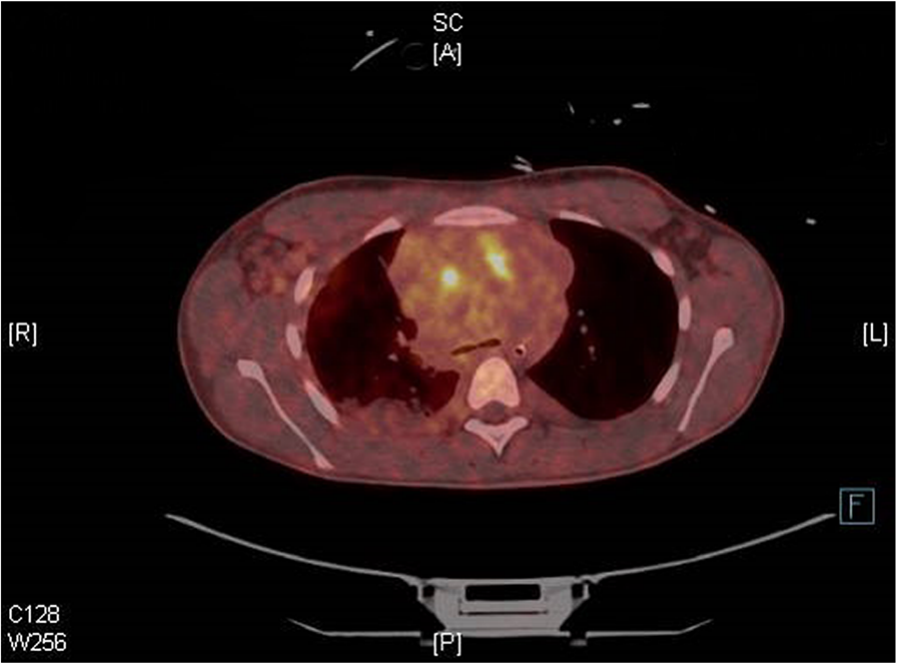
**Positron emission tomography computed tomography scan of mediastinum.** Increased activity equaling the mediastinal tumor mass.

The trauma computed tomography (CT) scan revealed multiple enlarged lymph nodes anteriorly in the mediastinum, bilaterally in the axillae, the throat, and along the aorta down to retroperitoneum. Furthermore, an almost collapsed superior vena cava was revealed (Figure 2). The following day we performed a positron emission tomography computed tomography scan. This revealed increased activity in the area equaling the lymphoid mass (Figure 3). On the suspicion of malignant lymphoma causing superior vena cava syndrome, the patient was transferred from the intensive care unit to the hematological service after 2 days, after he was stabilized and extubated. Excision of a cervical lymph node, a bone marrow biopsy, and analysis of pleural fluid for cytological examination were conducted. Biochemically, a lactate dehydrogenase of 350U/L and leukocytosis of 21.7×10^9^/L was found. His peripheral blood showed normal composition and his bone marrow showed no significant blast accumulation indicating no sign of T-cell lymphoblastic leukemia.

Cytological examination of his lymph node revealed an altered architecture and diffuse infiltration of tumor cells, with cell markers positive for CD3, CD5, CD2, CD7, CD4, Bcl-2, and TDT, and negative for CD23, Bcl-6, cyclin D1, CD34, CD8 CD56, tia-1, granzymB, and CD30, consistent with T–cell lymphoblastic lymphoma.

He started treatment immediately with vincristine and dexamethasone to reduce the tumor burden prior to the initiation of the chemotherapeutic regimen.

## Discussion

Reports of similar cases have been previously described. The focus of these have been on the diagnostic process [1], the management of pain and airway symptoms [1,3], differential diagnostic consideration [1,4], or unusual presentations of lymphoma [5,6].

Pietsch *et al*. [1] state how pleural fluid analysis results in a definitive diagnosis of lymphoma in five out of six children in case report series. The authors also emphasize the necessity of the correct treatment of the respiratory distress. The acute worsening of our patient’s respiratory condition after the draining of the pleural fluid was probably due to re-expansion edema, also described in two cases. It is seen after the drainage of large amounts of pleural fluid and is not a part of the lymphoma condition [1].

Yester and Ajizian describe the case of a 15-year-old boy and focus on alternative ways to provide analgesia during the invasive diagnostic procedures [3]. The differential diagnostic considerations have been described in relation to pneumonia in the case series by Pietsch *et al*. [1] and in a case report on a 14-year-old boy by Akbayram *et al*. [4].

Unusual presentations of the disease have been described in a case with a 20-year-old woman initially presenting with a large breast mass [5], and in the case of 2-year-old girl presenting with primary pleural lymphoma as the first and only manifestation of the disease [6], but no case has earlier been described with thoracic trauma as the main reason for seeking medical advice.

This trauma did not contribute to the pathogenesis and does not precipitate lymphoma However, the coexistence of a trauma and the underlying disease can blur the symptoms. This is particularly relevant as the affected group is often young adults where minor traumas are frequent. It is therefore important to acknowledge that the disease might not present itself with the usual symptoms.

## Conclusions

Our case report describes the diagnostic challenge when dealing with a patient suffering from T-cell lymphoblastic lymphoma. The symptoms are often vague and the condition often affects otherwise healthy young adults. In our case report, the disease was blurred by the chest trauma, offering a plausible explanation of the chest pain, and the protruding veins could have been misinterpreted as bruising from the trauma.

We emphasize the importance of reacting to an unexpected large quantity of pleural fluid compared to the trauma mechanism, and to consider underlying causes. Because this is a very aggressive type of lymphoma, it is crucial to ensure the correct diagnosis without delay. A delay of only a few days can be fatal. This case may interest doctors in all specialties that examine otherwise healthy young adults with chest pain.

## Consent

Written informed consent was obtained from the patient’s legal guardians for publication of this case report and accompanying images. A copy of the written consent is available for review by the Editor-in-Chief of this journal.

## Abbreviations

ALL: Acute lymphoblastic leukemia; BP: Blood pressure; CHOP: Cyclophosphamide doxorubicin vincristine and prednisolone; CT scan: Computed tomography scan; L: Litre; LDH: Lactate dehydrogenase; RF: Respiration frequency; SAT: Saturation.

## Competing interests

The authors declare that they have no competing interests.

## Authors’ contributions

AO wrote the manuscript. The patient was under the care of SB. AO and SB analyzed and interpreted patient data and SB contributed to revision of the manuscript. Both authors read and approved the final manuscript.
